# Long-term trends of Nitrogen oxides and surface ozone concentrations in Tehran city, 2002–2011

**DOI:** 10.1186/s40201-015-0218-7

**Published:** 2015-09-15

**Authors:** Saeed Motesaddi Zarandi, Mahmood Alimohammadi, Vahid Kazemi Moghaddam, Mohammad Sadegh Hasanvand, Mohammad Bagher Miranzadeh, Davarkhah Rabbani, Gholam Reza Mostafaii, Vali Sarsangi, Sajjad Hajiketabi, Ashraf Mazaheri Tehrani

**Affiliations:** Department of Environmental Health Engineering, School of Public Health, Shahid Beheshti University of Medical Sciences, Tehran, Iran; Department of Environmental Health Engineering, School of Public Health, Tehran University of Medical Sciences, Tehran, Iran; Student Research Committee, Sabzevar University of Medical Sciences, Sabzevar, Iran; Department of Environmental Health Engineering, School of Public Health and Air Pollution Research Center, Institute for Environmental Research, Tehran University of Medical Sciences, Tehran, Iran; Department of Environmental Health Engineering, School of Public Health, Kashan University of Medical Sciences, Kashan, Iran; Social Determinants in Health Promotion Research Center, Hormozgan University of Medical Sciences, Bandar Abbas, Iran; Student Research Committee, Jiroft university of medical science, Jiroft, Iran

**Keywords:** Tropospheric ozone, Daily changes, Long-term trends, Nitrogen oxides

## Abstract

**Background and aim:**

Tropospheric ozone is a problem with multi aspects - hazard to human health, plant, and welfare and a key factor to climate change, air pollution and atmosphere chemistry, as well. Behavior of ozone and nitrogen oxides (NO, and NO_2_) concentration is highly complex and variable; therefore, their trends as short and long-term were significantly attended. Most of the studies were carried out on the behavior of pollutant concentrations in North America, Europe, and East Asia, but few studies have been conducted in west Asia. The aim of this study was to assess daily changes and long-term trend of ozone and nitrogen oxides concentrations in Tehran city, Iran from March 2002 to September 2011.

**Material and methods:**

Data were collected from 18 air quality monitoring stations. The data were sorted as daily mean of 10 years (daily changes) and annual mean for each year (long-term trend). *One-sample test* was used to assess the statistical significance.

**Results:**

Current findings indicated that changes of ozone, NO, and NO_2_ concentrations are dependent from job shifts and photochemical reactions. Annual mean concentrations of ozone and NO_2_ were gradually increased, long-term trend of ozone and NO_2_ concentration indicated. The correlation between long term trend of ozone and NO_2_ was significant (*p* < 0.05).

**Conclusion:**

The controlling program was the most important factor in long-term concentration of ozone, and nitrogen oxides, but some problems and difficulties were accounted to perform controlling program.

## Introduction

Both advantages and disadvantages of ozone is based on height; as in stratosphere, the ozone protects human health, and other biological systems against harmful Ultra-violet (UV) radiation via absorbing UV, but in contrast the very gas could potentially damage human health, animal, and vegetables in surface of troposphere as a secondary pollutant [1–6]. Ozone is the second fatal pollutant following the particulate matter; also, and it has the essential role in making effects on human health. Approximately 0.7 million deaths are related to tropospheric ozone annually [7]. Ozone also affects crop yield of plants; it demolishes the *photosynthesis system* of plants as detriment about 14 to 26 billion dollars in 2000 [8].

Tropospheric ozone is formed during some chemical reactions amongst ozone precursors such as Hydrocarbons (HC_s_), and Nitrate Oxidants (NO_x_) [9]. The background concentrations of the ozone are strongly linked to its precursors’ concentrations [10] and the emission of precursors depends on sources mainly including gases produced by human and biological activities, but the human contribution including combustion, non-combustion, and agriculture is higher than biological activities [11]. Ozone is also strongly believed to play a key role in global warming, climate change, decliningair quality, and chemistry of atmosphere [12–14].

The levels of background ozone had an increasing trend in the northern hemisphere particularly in North America, Europe, Eastern and Southeastern Asiain recent century, but recently this increase is controlled even in some regions with decreasing trend due to controlling programs [15–17].

This behavior of ozone and its precursors were considered in many literatures since the precursors of tropospheric ozone mainly affects the trends of ozone [10, 17]. Lal *et al.* [18] reported that daily variation of the concentration of the tropospheric ozone was related to boundary layer processes, and local wind direction pattern, the high concentration in background ozone was observed in the seasons, fall and winter; also, this higher concentration was due to excessive production of ozone processors resulted in high traffic weight loads, and lower boundary layer heights in the mentioned seasons [18]. Daily changes of background ozone concentrations investigated by Xu *et al.* [19] indicated an increased tropospheric ozone [19].

Production and trend of ozone are affected by regulation of controlling programs. Consequently, background levels of ozone could be controlled by regulating an appropriate policy.

It should be noted that controlling programs could not alwaysbe effective; the main factor of ozone products is not a manmade source and biological factors should occasionally be considered. In controlling programs that were applied in the United States, there was no acceptable influence in NO_x_-reduction program in background ozone reduction as NO_x_ reduction in the emissions’ rate per mile compensated with increase of travel miles; as a result, target pollutant for controlling programs were changed from NO_x_to Volatile Organic Components (VOCs) in some regions [20].

In some studies, long-term changes of ozone and its precursors were assessed [21–23]. Considering long-term trends of surface ozone are important to understand effects of this matter on human health, plants, and climates and for selecting air pollution control programs (as mentioned above), particularly in large scale.

Richter *et al.* [24] demonstrated that trends of ozone precursors were increased over the last decades particularly in China; in contrast with the United States and Europe, producing trend of tropospheric ozone and its precursors were dropped [21, 24].

Some of the studies were performed in continentalscale. In Europe, a study conducted by Jonson *et al.* [22] indentified that the ozone concentration was raised in winter and declined in summer, and also determined that changes in ozone is reasonably not correlates with changes in NO_x_ [22].

In a systematic review it was showed that changes in ozone trends had a heterogeneous distribution so that in 22 stations, a rising trend was reported, in eight was reversed, and in some stations no change was observed [15].

More studies should be conducted forozone and its own precursors’ concentrations to survey the mentioned trends. The aim of the current study was to survey diurnal cycles and long-term trends for these pollutants in Tehran, Iran from March 2002 to September 2011, while more stations for long-term measurement of ozone and its precursors located in North America, Europe and East Asia [25, 26].

## Material and methods

**Fig. 1:**
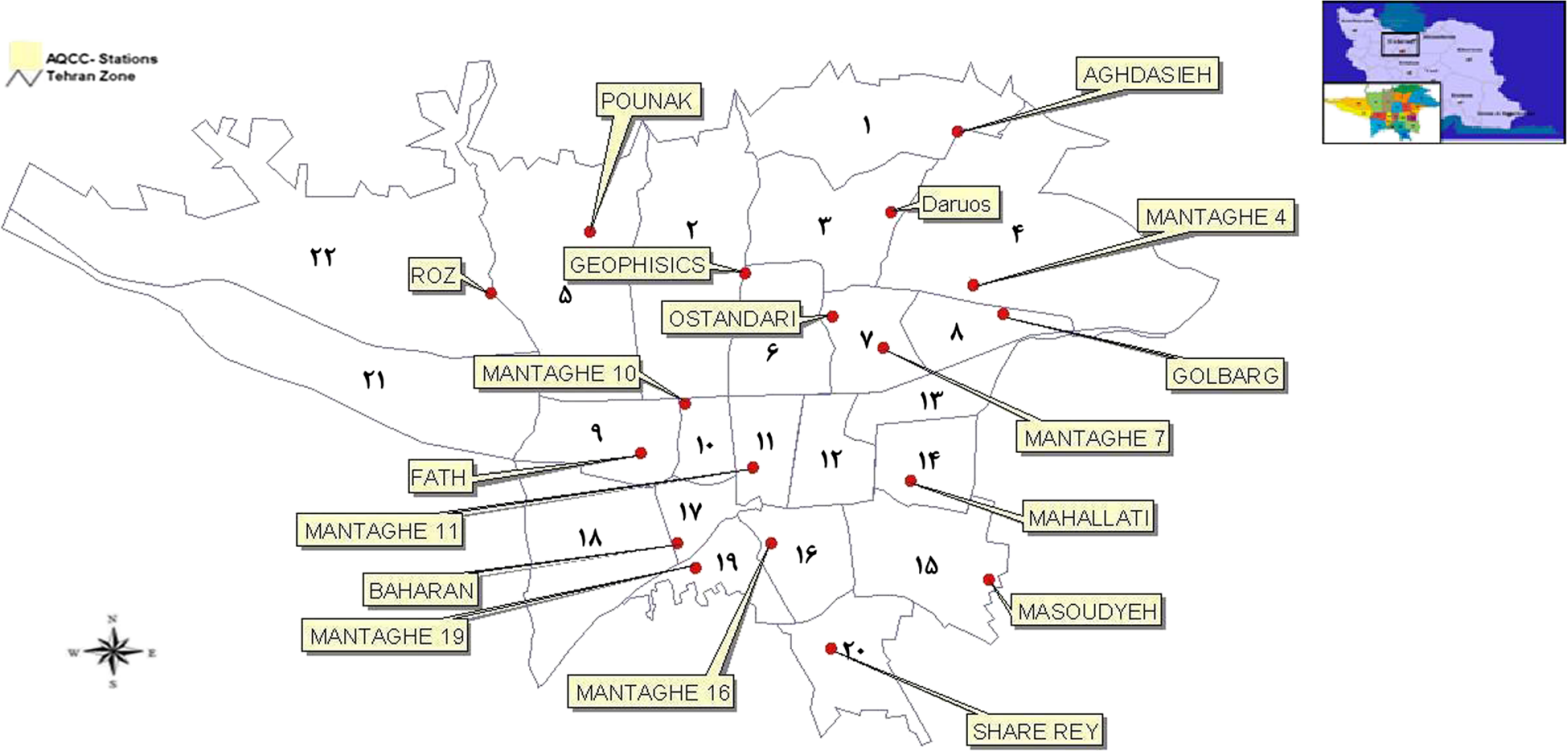
Location of air quality monitoring stations (Air Quality Control Company)

All of the data were collected from 18 regional air monitoring stations in Tehran city. They were given from Air Quality Control Companyof Tehran. Tehran had significantly a growth in urbanization and industry during the last years, [27]. The stations automatically recorded the concentrations from March 2002 to September 2011. The locations of these stations are illustrated (Fig. 1). The measuring method for NO_x_ (NO_2_ and NO) concentration was Chemical Luminsans and Non-dispersive Infrared Sensor (NDIR), O_3_ Analyzerwere used to measure the O_3_ concentration. The values of the concentration of O_3_, and NO_x_ (NO_2_ and NO) were measured, hourly. The average of hour-values was taken and the mean of 10 years’ days considered as daily changes. Also, the average was taken of daily means during a year, this considered asannual mean (long-term) concentration. To determine the statistical significance, *one sample test* was used. The significant level of greater than 0.05 was used to evaluate the ozone and NO_2_ behaviors in long-term trends. We removed NO concentration from long-term trend due to the high behavioral similarities to NO_2_.

## Results and discussion

The aims of this study were to investigate diurnal changes and annual trend for tropospheric ozone and the concentration of its main precursors in Tehran city during adecade (2002 to 2011). We focused on background levels of these pollutants collected from regional air monitoring stations across all of the areas.

**Fig. 2:**
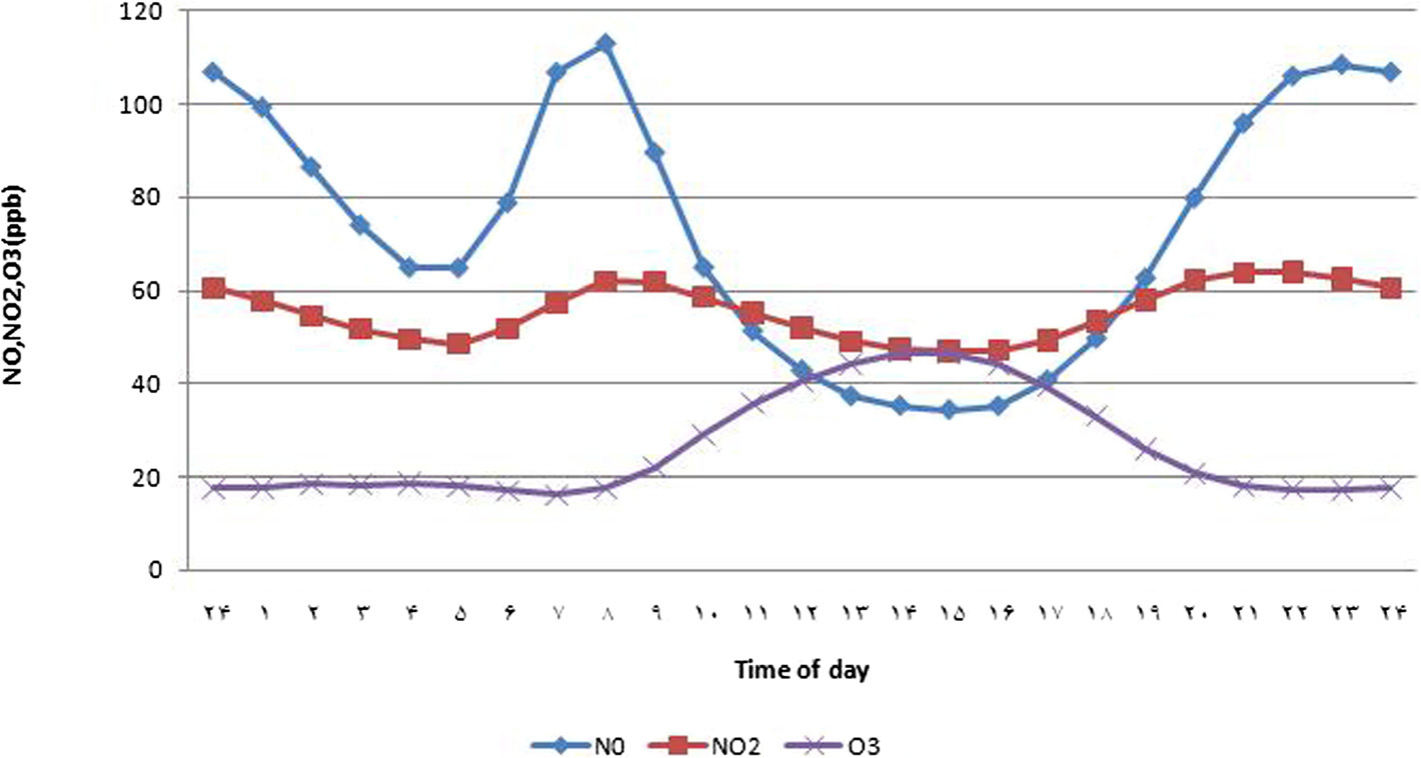
Diurnal variations in ozone, NO and NO_2_ concentration from March 2002 to September 2011

**Fig. 3:**
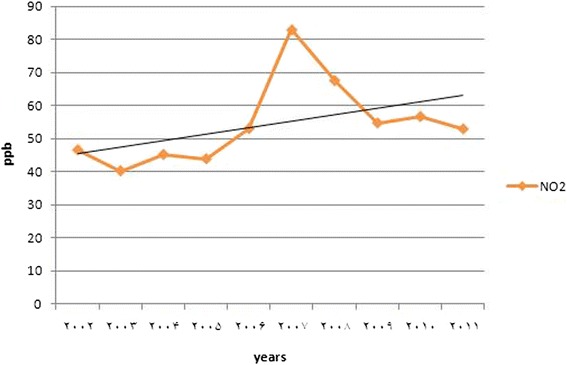
Annual trend of NO_2_ mean concentration during 2002 to 2011

**Fig. 4:**
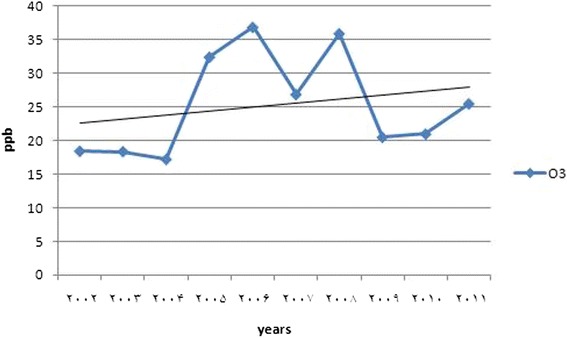
Long-term trend of O_3_ concentration during 2002 to 2011

The daily changes of ozone, NO and NO_2_ concentrations from 2002 to 2011 were shown (Fig. 2 and Table 1), and the annual trend of pollutants are illustrated (Figs. 3 and 4).

**Table 1:** A summary statistic table for ozone, NO and NO_2_ concentration according to time of day

Time	Pollutant											
	NO				NO_2_				O_3_			
	Min	Max	Mean	SD	Min	Max	Mean	SD	Min	Max	Mean	SD
1	74.81	150.17	99.27	25.34	41.16	74.04	57.87	11.15	7.64	41.23	17.66	10.72
2	67.18	131.47	86.45	21.21	37.51	70.14	54.56	10.70	8.34	42.02	18.47	10.84
3	54.97	113.36	73.96	18.68	34.10	66.25	51.65	10.24	8.83	34.24	18.38	9.11
4	48.15	99.18	64.81	16.84	34.46	62.38	49.61	9.16	9.16	32.38	18.61	8.83
5	49.41	90.62	64.74	14.46	32.42	61.06	48.53	8.79	8.70	31.24	17.98	8.31
6	57.13	104.05	78.77	15.20	38.08	62.89	51.90	7.71	7.60	30.73	17.06	8.34
7	80.77	140.21	106.68	19.26	44.94	69.87	57.41	8.75	7.06	33.29	16.24	8.71
8	78.66	180.15	112.90	33.85	49.23	79.75	61.98	10.28	8.35	37.33	17.52	9.74
9	51.82	165.96	89.58	35.78	48.14	80.51	61.81	9.98	12.30	37.02	22.05	9.09
10	35.62	118.02	64.84	24.97	43.84	76.77	58.52	10.10	17.37	42.78	28.95	8.89
11	26.15	86.73	51.29	18.07	38.74	70.22	55.26	10.30	24.12	50.66	35.73	9.48
12	20.77	69.17	42.93	14.27	32.89	66.57	51.94	10.96	29.82	58.69	40.64	10.10
13	17.92	58.83	37.30	11.58	32.88	61.35	49.07	9.63	33.40	63.30	44.20	10.83
14	16.38	52.16	35.22	10.75	27.93	58.83	47.49	9.48	35.80	66.03	46.73	11.60
15	15.97	50.18	34.38	9.80	30.22	58.85	47.02	8.86	34.24	66.26	46.52	11.56
16	17.68	52.78	35.19	10.07	30.79	60.57	47.11	9.14	31.27	63.82	44.26	11.32
17	21.47	60.45	40.72	30.02	31.81	61.33	49.19	9.85	26.46	56.35	39.42	11.22
18	29.31	71.32	49.67	14.70	34.93	66.63	53.44	9.37	20.65	47.11	33.02	9.24
19	43.03	86.55	62.56	18.19	41.00	70.58	57.97	9.04	13.93	38.91	25.95	8.96
20	57.14	109.93	79.76	21.07	47.13	74.31	62.16	8.83	9.40	39.39	20.75	9.94
21	69.60	133.01	95.89	22.01	49.17	77.45	63.68	9.84	7.88	41.33	18.00	10.52
22	74.46	148.28	105.93	24.26	48.86	78.44	63.83	10.84	7.43	42.46	17.37	10.96
23	77.43	156.65	108.41	25.64	46.84	77.82	62.59	11.19	7.27	42.84	17.29	11.01
24	78.44	158.66	106.77	25.70	44.78	76.74	60.54	11.22	7.24	42.86	17.52	11.17

### Diurnal variations

The concentration of NO had two peaks at 7 to 8 A.M (~110 ppb) and 9 to 12 P.M (~105 ppb). Moreover, the NO_2_ peak occurred around 8 to 9 A.M (~61 ppb) (following NO peak) and at 8 to 11 P.M (~63 ppb). Conversely, a peak was observed in the concentration of ozone in the middle of the day reached to its peak at 1 to 3 P.M and the turning point of the levels of ozone occurred around 2 P.M (~50 ppb). Coincides with the maximum level of ozone in middle of the day, minimum levels of NO and NO_2_ was observed (~35 and 50 ppb, respectively).

In other words, the opposite behavior of ozone was demonstrated in comparison with its processors (NO and NO_2_) in terms of mean daily change. As maximum levels of ozone illustrated in noon, minimums were in the morning and night, in contrast, there was a maximum level for NO and NO_2_ in the morning and night and minimum during noon. These events are resulted from photochemical reactions of NO, and NO_2_ during rush hours and also job shifts. The rapid increase of NO concentration correlates with beginning of job hours in the morning. The peak of NO_2_ concentration related to converting of NO to NO_2_, over 1 to 2 h. With the rising of sun, the photochemical reactions of NO and NO_2_caused appearing of O_3_. The concentration of O_3_ would be in its own maximum level in the middle of the day. These results are compatible with some studies [19, 23, 28].

### Long-term trends

Annual trends of ozone and the concentration of its main precursor were portrayed (Figs. 3 and 4). Despite the increase in the concentration of NO_2_ in all years from 2002 to 2011, some changes were demonstrated in this period; the levels of NO_2_was decreased in 2002 to 2003, then it was almost constant to 2005, but in 2005 to 2007 (maximum level) it was increased, afterwards it was decreased in 2009 then formed astraight line (Fig. 3).

These trends could relate to controlling program (entering of new equipped vehicles with better fueling systems, using of catalytic converters in new vehicles, and having compulsory standards of Euro 1 and 2 for under-constructing ones). In spite of increased levels of NO_2_ concentration, very small increases were observed. Although, controlling program in Tehran city was considered to use engines with better combustion and produce fewer NO_2_, and decreasing emission of NO_2_ per mile, the number oftraveled miles increased as NO_2_ continues to rise, some of the studies [23, 29, 30] confirm this finding.

According to the statistical analyzes (one-sample test), changing trends of ozone concentration was linked to NO_2_ concentration (*P* > 0.05) and similar to the majority of findings identified that the main factor of reducing concentration of ozone is NO_2_ [23, 31]; a correlation was obtained between the ozone and NO_2_ concentration, some studies [23, 32] are consistent with gained findings, but are not compatible with the results of Jonson *et al.* [22].

The trend of surface ozone had a fluctuating process. The two peaks were indicated in 2005 and 2007. The trend of the concentration of the ozone was a gentle increasing in total period (for more details see the Fig. 4).

It is seemed that, small rate of ozone increase is due to controlling programs of air pollution, particularly NO_2_. Although a controlling program is being conducted in Tehran, like other developed countries, there is a gap between plan and implementation [33].

It should be considered that our data are collected from interurban stations and part of reported concentration might be resulted from industries around the city, but the same data are very valuable for the detection of pollutants behavior. It is also noted that evaluation of ozone trends are very difficult since these trends have many inter annual variations and plenty of time is required to assess.

## Conclusion

This study targeted the short and long-term changes of ozone and the concentration of its precursors. It determined that job shifts and solar radiation and photochemical reactions are the main causes of daily changes for these pollutants; in case of long-term trend of NO_2_, it was the main factor for ozone trend. Based on the current findings, giving more attention to high impact of job shifts on daily changes of pollutants is necessary, legislation and facilities should be adopted to improve and move towards the use of public transportation systems. In addition, the public transportation system should be extended and reconstructed. The effective actions were performed to improve fuel that all of the activities should progressively be continued.

More researches should be performed to focus on the impact of industrial emission on ozone behavior, using of treated data for the same studies, and evaluating the effects of controlling program stages on pollutants behavior, as well.

## References

[1] Karnosky DF, Skelly JM, Percy KE, Chappelka AH (2007). Perspectives regarding 50 years of research on effects of tropospheric ozone air pollution on US forests. Environ Pollut.

[2] Ren W, Tian H, Liu M, Zhang C, Chen G, Pan S, Felzer B, Xu X (2007). Effects of tropospheric ozone pollution on net primary productivity and carbon storage in terrestrial ecosystems of China. J Geophys Res Atmos (1984–2012).

[3] Ariyaphanphitak W, Chidthaisong A, Sarobol E, Bashkin V, Towprayoon S (2005). Effects of elevated ozone concentrations on Thai jasmine rice cultivars (Oryza sativa L.). Water Air Soil Pollut.

[4] Seinfeld J (1991). Rethinking the Ozone problem in urban and regional air pollution.

[5] Norval M, Lucas R, Cullen A, De Gruijl F, Longstreth J, Takizawa Y, Van Der Leun J (2011). The human health effects of ozone depletion and interactions with climate change. Photochem Photobiol Sci.

[6] Staehelin J, Harris N, Appenzeller C, Eberhard J (2001). Ozone trends: A review. Rev Geophys.

[7] Anenberg SC, Horowitz LW, Tong DQ, West JJ (2010). An estimate of the global burden of anthropogenic ozone and fine particulate matter on premature human mortality using atmospheric modeling. Environ Health Perspect.

[8] Van Dingenen R, Dentener FJ, Raes F, Krol MC, Emberson L, Cofala J (2009). The global impact of ozone on agricultural crop yields under current and future air quality legislation. Atmos Environ.

[9] Jacob DJ (2000). Heterogeneous chemistry and tropospheric ozone. Atmos Environ.

[10] Frost G, McKeen S, Trainer M, Ryerson T, Neuman J, Roberts J et.al. Effects of changing power plant NOx emissions on ozone in the eastern United States: Proof of concept, J. Geophys. Res. 111, D12306, doi:10.1029/2005JD006354.

[11] Ohara T, Akimoto H, Kurokawa JI, Horii N, Yamaji K, Yan X, Hayasaka T (2007). An Asian emission inventory of anthropogenic emission sources for the period 1980–2020. Atmos Chem Phys.

[12] Johnson C, Henshaw J, Mclnnes G (1992). Impact of aircraft and surface emissions of nitrogen oxides on tropospheric ozone and global warming.

[13] Mohnen V, Goldstein W, Wang W-C (1993). Tropospheric ozone and climate change. Air Waste.

[14] Fishman J, Ramanathan V, Crutzen P, Liu S (1979). Tropospheric ozone and climate. Nature.

[15] Vingarzan R (2004). A review of surface ozone background levels and trends. Atmos Environ.

[16] Oltmans S, Lefohn A, Harris J, Galbally I, Scheel H, Bodeker G, Brunke E, Claude H, Tarasick D, Johnson B (2006). Long-term changes in tropospheric ozone. Atmos Environ.

[17] Cooper OR, Gao RS, Tarasick D, Leblanc T, Sweeney C (2012). Long‐term ozone trends at rural ozone monitoring sites across the United States, 1990–2010. J Geophys Res Atmos (1984–2012).

[18] Lal S, Naja M, Subbaraya B (2000). Seasonal variations in surface ozone and its precursors over an urban site in India. Atmos Environ.

[19] Xu X, Lin W, Wang T, Yan P, Tang J, Meng Z, Wang Y (2008). Long-term trend of surface ozone at a regional background station in eastern China 1991–2006: enhanced variability. Atmos Chem Phys.

[20] Ryerson T, Trainer M, Holloway J, Parrish D, Huey L, Sueper D, Frost G, Donnelly S, Schauffler S, Atlas E (2001). Observations of ozone formation in power plant plumes and implications for ozone control strategies. Science.

[21] He H, Stehr J, Hains J, Krask D, Doddridge B, Vinnikov K, Canty T, Hosley K, Salawitch R, Worden H (2013). Trends in emissions and concentrations of air pollutants in the lower troposphere in the Baltimore/Washington airshed from 1997 to 2011. Atmos Chem Phys Discuss.

[22] Jonson J, Simpson D, Fagerli H, Solberg S (2006). Can we explain the trends in European ozone levels?. Atmos Chem Phys.

[23] Ding A, Wang T, Thouret V, Cammas J-P, Nédélec P. Tropospheric ozone climatology over Beijing: analysis of aircraft data from the MOZAIC program. Atmos Chem Phys. 2008;8.

[24] Richter A, Burrows JP, Nüß H, Granier C, Niemeier U (2005). Increase in tropospheric nitrogen dioxide over China observed from space. Nature.

[25] Oltmans S, Lefohn A, Scheel H, Harris J, Levy H, Galbally I, Brunke EG, Meyer C, Lathrop J, Johnson B (1998). Trends of ozone in the troposphere. Geophys Res Lett.

[26] Naja M, Akimoto H (2004). Contribution of regional pollution and long‐range transport to the Asia‐Pacific region: Analysis of long‐term ozonesonde data over Japan. Atmos Chem Phys Discuss (1984–2012).

[27] Afrakhteh H (2003). Urban Growth and Experience of New Town Planning in the Developing Countries: A Case Study of Tehran Metropolis [J]. Urban Planning Overseas.

[28] Ibarra-Berastegi G, Madariaga I, Elías A, Agirre E, Uria J (2001). Long-term changes of ozone and traffic in Bilbao. Atmos Environ.

[29] Wang T, Wei X, Ding A, Poon C, Lam K, Li Y, Chan L, Anson M (2009). Increasing surface ozone concentrations in the background atmosphere of Southern China, 1994–2007. Atmos Chem Phys Discuss.

[30] Beard BA, Freas WP. National Air Quality and Emissions Trends Report. Environ Stat Assessment Forecasting. 1994;163.

[31] Gimeno L, Hernández E, Rúa A, García R, Martín I (1999). Surface ozone in Spain. Chemosphere.

[32] Safieddine S, Clerbaux C, George M, Hadji‐Lazaro J, Hurtmans D, Coheur PF, Wespes C, Loyola D, Valks P, Hao N (2013). Tropospheric ozone and nitrogen dioxide measurements in urban and rural regions as seen by IASI and GOME‐2. Atmos Chem Phys Discuss.

[33] Atash F (2007). The deterioration of urban environments in developing countries: Mitigating the air pollution crisis in Tehran, Iran. Cities.

